# Spontaneous Goal-Related Cognition Predicts Well-Being and Real-World Goal Attainment Across Adulthood in a Think-Aloud Study

**DOI:** 10.21203/rs.3.rs-10102477/v1

**Published:** 2026-07-11

**Authors:** V. A. Puig Rivera, Q. Raffaelli, K. Chambers, G. Oettingen, J. J. Arch, M. D. Grilli, J. R. Andrews-Hanna

**Affiliations:** 1 Department of Psychology, The University of Arizona, Tucson, AZ 85721, USA; 2 Department of Psychology, University of Calgary, Calgary, AB T2N 1N4, Canada; 3 Department of Psychology, New York University, New York, NY 10003, USA; Department of Political and Social Sciences, Zeppelin University Friedrichshafen, Friedrichshafen, Germany; 5 Department of Psychology and Neuroscience, University of Colorado Boulder, Boulder, CO 80309, USA; 6 Evelyn F. McKnight Brain Institute, The University of Arizona, Tucson, AZ 85719, USA; 7 Department of Cognitive Science, The University of Arizona, Tucson, AZ 85721, USA

## Abstract

Decades of research has established that how we think about our goals shapes psychological well-being and daily behavior, yet much of what we know derives from paradigms that deliberately prompt goal cognition. Across two studies (*N* = 173), we developed and applied a novel hierarchical coding scheme to Think-Aloud Protocol recordings to understand whether these same processes emerge spontaneously in the stream of consciousness, whether they carry meaningful consequences for real-world behavior, and how they change in older age. We quantified participants’ spontaneous engagement in mental contrasting (MC), a self-regulatory strategy that links desired futures with present obstacles to facilitate solutions that promote effective goal pursuit, in the absence of instruction or prompting. In Study 1 (*n* = 77 young adults), higher depression and anxiety predicted less frequent spontaneous mental contrasting (MC3), while higher trait mindfulness predicted more frequent MC3 and less dwelling on obstacles. Study 2 (*n* = 95 younger and older adults) largely replicated and extended these findings, further revealing that despite a similar number of goal-related thoughts, older adults produced a higher proportion of MC3 thoughts relative to younger adults. Moreover, more frequent use of mental contrasting when spontaneously thinking about one’s goals predicted significantly greater goal progress and accomplishment over time, independent of age. These findings demonstrate that individuals spontaneously vary in the depth of goal-related elaboration during spontaneous thought, that these variations associate meaningfully with wellbeing and real-world goal success, and that spontaneous engagement in solution-focused goal elaboration seems to differ across the adult lifespan.

## Introduction

Achieving one’s goals and ambitions is a cornerstone of human psychological health and functioning. Within contemporary research, the capacity to set and pursue meaningful goals is recognized as fundamental to subjective well-being and life purpose^1,2,3,4,5^. Goal achievements similarly contribute to overall life satisfaction, positive affect, and psychological growth^6,7,8,9^. However, the relationship between goal pursuit and mental health extends beyond mere attainment; it also depends on the nature and quality of how goal-related thoughts occupy and organize the mind^10^.

Dysfunctional patterns of goal setting and pursuit have been identified in mental health difficulties^8,11,12,13^. Major depressive disorder is characterized by conditional goal setting^14^, in which personal goals become tightly linked to future identity and self-worth. Depressed individuals also anticipate less pleasure from goal attainment, less likelihood of goal attainment, and greater distress following failure^15–16^. Depression has also been linked to having fewer approach goals^11^. Anxious individuals often endorse more avoidance goals, generate more avoidance plans, fewer approach plans, and are less specific when framing approach goals^12,13,17^. Anxiety also shapes mental simulation through catastrophizing about non-attainment, producing simulations that heighten negative affect^17^ and overgeneralization^12^.

In contrast, mindfulness potentially represents a protective factor by facilitating adaptive goal pursuit. Defined as voluntary, non-judgmental attention to present-moment experience^18,19^, mindfulness is thought to support goal selection by promoting self-concordant goals^20,21^ and similarly supports pursuit by enhancing persistence and goal internalization^20^. Mindfulness further strengthens emotion regulation through enhanced emotional granularity^22,23^ and decentering, enabling flexible adjustment following setbacks^24,25, 26^. Despite the present-oriented focus often linked to mindfulness, mindful individuals may adopt this heightened attentional awareness and specificity to the way they recall past experiences and hypothetical future events^27^. Thus, this capacity for clear, granular mental simulation may extend naturally to the goals present in one’s mind and facilitate a detailed awareness of possibilities that serve effective goal pursuit.

### Goals Across the Lifespan

Lifespan developmental theories provide compelling frameworks suggesting that emotional regulatory capacities and motivational processes undergo significant transformations as individuals’ goals, resources, and time perspectives change with age^28,29^. Socioemotional Selectivity Theory (SST)^30^ posits that as people age and perceive their future time horizons as increasingly limited, a fundamental shift occurs in goal priorities. Specifically, older adults systematically prioritize emotionally meaningful goals and the deepening of social connections over long-term instrumental goals and social network expansion behavior that characterize younger adulthood^31^. This shift is seen as a response to the recognition of finite remaining time, leading to a focus on goals that provide emotional meaning and satisfaction rather than those oriented toward uncertain future payoffs^32^. Complementing this perspective, the Selection, Optimization, and Compensation model (SOC)^33^ suggests that healthy aging involves strategic allocation of increasingly limited resources toward a carefully selected subset of meaningful, attainable goals^34^.

From a cognitive perspective, older adulthood has been characterized by a transition from exploratory search toward exploitation of accumulated knowledge and routines^35^. Whereas younger adults emphasize growth and gains, older adults often prioritize maintenance and loss prevention^2,36^. Importantly, this shift is suggested to reflect adaptation rather than decline mechanisms, as older adults report higher well-being and perform more effectively when pursuing maintenance-framed goals^3^. Together, these findings hint at a motivational shift toward safeguarding existing capabilities in later life.

### Mental Contrasting as Self-Regulation

A central theoretical framework for understanding effective and adaptive goal pursuit is Mental Contrasting (MC)^37^, a sophisticated self-regulatory strategy within Fantasy Realization Theory (FRT)^38,39^. MC involves actively searching for and vividly imagining both the desired future outcome and the critical obstacle of present reality that stands in its way. When expectations of success are high, MC energizes goal pursuit; when expectations are low, it facilitates adaptive disengagement, allowing individuals to redirect resources toward more promising endeavors.

Experimental research demonstrates that instructed MC facilitates improved academic and creative performance^39,40^, successful health behavior changes^41^, more effective time management^42^, and enhanced interpersonal goal attainment^37^. The effects appear to rest on MC’s capacity to transform vague wishes into specific goals and actionable plans with subsequent adaptive action^39,43^. By contrast, merely indulging in fantasies without considering obstacles, or dwelling on difficulties without envisioning desired end states, represents incomplete goal-related thought, as they fail to establish critical associative links between the desired future outcome and the present reality as an obstacle, as well as between the obstacle and the behavior needed to overcome it^44,45^, often resulting in indiscriminate goal pursuit and wasted effort.

### Goals and Spontaneous Thought

Despite the robust experimental evidence supporting MC as an effective self-regulatory strategy, a key gap remains. The vast majority of MC and related research on mental simulation has relied on paradigms in which participants are explicitly instructed or prompted to engage in specific modes of goal-related thought. In typical MC experimental protocols, researchers direct participants to find and imagine their desired future outcome in detail, to then identify the critical obstacle(s) in their present reality, and finally to generate plausible solutions to the obstacle. While these instructional paradigms have been invaluable for establishing the efficacy of MC, they leave fundamental questions unanswered: (1) How often do individuals spontaneously engage in MC during naturally occurring, unprompted periods? (2) How does the use of spontaneous MC in unprompted contexts relate to individual differences in well-being? (3) Does spontaneous MC predict real-world goal attainment? And (4) Do these relationships differ between younger and older adults?

Substantial research establishes that spontaneous thought content often relates to goal pursuits^46,47,48,49,50,51,52^, yet the critical focus of this present work is not whether people spontaneously think about their goals, but *how* they think about them. Understanding the natural structure and quality of spontaneous goal-related cognition may provide important insights into how individuals successfully pursue and attain their goals in day-to-day contexts.

A handful of studies have begun to address this gap by examining more spontaneous manifestations of goal-related cognition^53,54^. However, these studies have “taught” participants how to engage in these self-regulatory modes of thought or prompt participants’ attention toward goals. Beyond implications for theories of goal-cognition, spontaneous thought, and aging, uncovering whether the hierarchical levels of goal elaboration described in FRT emerge spontaneously in the unstructured stream of consciousness^55^, whether they hold links with real-life goal progress and mental health, and quantifying how these processes change with age could offer novel markers of disease-related decline and intervention success.

### The Present Research

The present research addresses these gaps by developing and applying a novel scoring procedure to measure spontaneous MC within unprompted thoughts captured via the Think-Aloud Protocol (TAP)^56,57^. Rather than instructing any form of goal-related thinking, this approach asks participants to verbalize whatever enters awareness during an unstructured 10-minute period^58,59^. Study 1 establishes individual-difference correlations between spontaneous MC and psychological well-being (depression, anxiety, and trait mindfulness) in 77 young adults. Study 2 (preregistered on OSF)^60^ aimed to replicate these relationships in an independent younger and older adult sample, test an additional hypothesis that spontaneous mental contrasting would predict real-world goal progress and uncover age differences in spontaneous MC. For a detailed outline of our pre-registered hypotheses, see Supplementary Information S1.

## Study 1

### Sample and Procedures

#### Participants

This sample consisted of 77 young adults (43 F, 33 M, 1 non-binary; ages 17–28 years, *M* = 19.53, *SD* = 2.34) recruited from the University of Arizona in exchange for course credit or cash payment. Written informed consent was obtained from all participants, and all procedures were approved by the University of Arizona’s Institutional Review Board. The sample was racially diverse, identifying predominantly as Non-Hispanic White (*n* = 35), Latine/Hispanic (*n* = 25), and as other racial or ethnic groups or preferring not to answer (*n* = 17). A detailed demographic breakdown is available in Supplementary Information Table S5.

### Scales and Measures

#### Trait-level measures

Depression, anxiety, and trait mindfulness were assessed using the PHQ-9^61^, GAD-7^62^, and MAAS^18^, respectively; full scale descriptions and scoring thresholds are provided in the Supplementary Information. Study 1 participants reported mild depressive symptoms (M = 4.96, SD = 4.32 on the PHQ-9) on average, mild anxiety (M = 4.80, SD = 4.45 on the GAD-7), and moderate-to-high trait mindfulness (M = 4.04, SD = 0.84 on the MAAS).

#### Think Aloud Protocol

We used a Think-Aloud Protocol (TAP) to capture participants’ stream of consciousness. The TAP offers a valuable window into thought as it naturally unfolds without experimenter direction or constraint^63^. Unlike laboratory-based tasks that typically detect task-unrelated thought during ongoing activity or prompted goal-elicitation, shifting direct attention toward goals, TAP captures the ongoing phenomenology of unprompted thought.

**Fig. 1.**
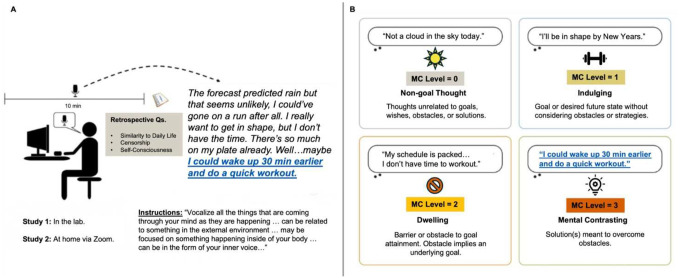
Mental Contrasting coding scheme and study paradigm. (**A**) Participants completed a 10-minute think-aloud task in which they verbalized their stream of consciousness. Retrospective questions assessed similarity to daily life and censorship. The paradigm was administered in-lab (Study 1) and remotely via Zoom (Study 2). (**B**) A four-level coding scheme was adapted from Oettingen (2000) and used to classify spontaneous goal-related cognition during the Think-Aloud Protocol. Example quotes are illustrative.

Participants in Study 1 sat alone in a testing room for 10 minutes, without access to their phones, while their continuous verbalizations were audio recorded (Fig 1). Consistent with prior research using unprompted TAP procedures^64^, participants received no direction regarding what to think about. The testing environment provided minimal external stimulation. Instructions specified that participants should continuously verbalize whatever came to mind, including internal thoughts or mental images, external stimuli, and bodily sensations or feelings (e.g., discomfort, pain, hunger). Detailed procedures can be found in the Supplementary Information.

Following the TAP session, participants completed post-task questions to assess the ecological validity of their verbalizations. On average, participants reported low censorship of their thoughts (*M* = 2.09, *SD* = 0.91, scale: 1–5), moderate self-consciousness during the task (*M* = 2.80, *SD* = 1.29), and high similarity of their verbalizations to typical day-to-day thinking (*M* = 3.89, *SD* = 0.93; all reported on a 1–5 scale, 1 = *not at all*, 5 = *extremely*). These procedures and values are consistent with those reported in Raffaelli et al.^59^; instructions and inter-rater statistics are provided in the Supplementary Information.

#### Mental Contrasting Scoring

To systematically quantify the depth and sophistication of goal-related processing in naturally occurring thought, we developed a four-level hierarchical coding scheme that classifies spontaneous cognition along a continuum from non-goal content to fully integrated mental contrasting (MC0 to MC3). This coding procedure represents a novel methodological contribution, allowing researchers to apply the well-established theoretical constructs of FRT^38^ to unprompted, ecologically valid streams of thought captured through the TAP, thereby bridging gaps between laboratory experiments of instructed mental contrasting and the spontaneous cognitive processes that characterize real-world goal pursuit.

Critically, the coding levels are incremental and mutually exclusive. Higher levels contain elements from previous levels, whether explicitly stated or implied. For example, mentioning an obstacle to a goal necessarily implies the presence of a goal, even if not explicitly articulated. Each thought unit receives the score corresponding to the highest level reached within that thought, ensuring that the most sophisticated level of goal elaboration present is captured. Fig. 1 provides descriptions and examples of each level.

Two independent raters coded all participant thoughts using a 0–3 Mental Contrasting scale. Following standardized training, raters achieved excellent inter-rater reliability (κ*w* = 0.804, *p* < .001; *z* = 38.78, *p* < .001)^65^. Final scores for each thought were calculated as the mean of both raters’ scores, producing values from 0 to 3. These thought-level scores were then aggregated to the participant level by calculating the proportion of thoughts falling into each mental contrasting category (MC0-MC3). These participant-level metrics served as outcomes in subsequent regression analyses examining associations with depression, anxiety, and mindfulness predictors. See Supplemental Methods for detailed procedures.

### Results

#### Data Analysis Procedures

Depression (PHQ-9) and anxiety (GAD-7) variables were log-transformed using a log(1 + x) transformation prior to analysis due to positive skewness. Due to continued evidence of non-normal residual distributions (Shapiro-Wilk *p* < .01) after log transformation, all regression analyses employed robust linear regression. Models were estimated using the Robustbase package^66^ with significance testing conducted via the lmtest package^67^ in R^68^. Given the well-documented co-occurrence of depression and anxiety symptoms, we examined collinearity between these predictors prior to primary analyses. Results of these analyses for Study 1 and 2, including joint correlations isolating the unique contribution of each predictor, are reported in Supplementary Information S2.

#### Characteristics of Spontaneous Goal Cognition

On average, participants produced 28.9 thoughts (*SD* = 14.2, range: 5–63) during the 10-minute TAP (Table 1). Approximately 24.1% of participants’ thoughts were goal-related (*M* = .24, *SD* = .17), a proportion significantly greater than zero (*t*(76) = 12.73, *p* < .001). Goal-related thoughts were predominantly indulging in nature (MC1: M = 15.6%).

#### Individual Differences in Relation to Mental Contrasting and Psychological Well-Being

**Fig. 2.**
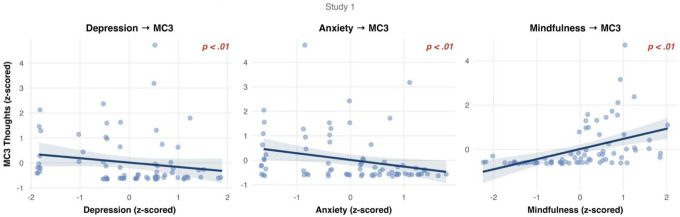
Relationships Between Well-Being and Spontaneous Goal Cognition (Study 1). Points represent individual participants. Lines reflect robust linear regression fits with 95% CI shading. Annotations show significance level.

Robust linear regression analyses examined associations between the proportion of spontaneous MC thoughts and trait measures of mindfulness (MAAS), depression (PHQ-9), and anxiety (GAD-7). Primary analyses focused on relationships between trait metrics and MC3. Higher depression and anxiety symptoms significantly predicted a lower proportion of MC3 thoughts (depression: β = −0.16, *SE* = 0.05, *z* = −3.00, *p* = .003; anxiety: β = −0.13, *SE* = 0.05, *z* = −2.78, *p* = .005; Fig. 2). Conversely, higher trait mindfulness predicted a greater proportion of MC3 thoughts (β = 0.22, *SE* = 0.04, *z* = 5.48, *p* < .001; Fig. 2). Exploratory analyses extended findings beyond MC3 thoughts to examine associations between wellbeing and the proportion of dwelling thoughts (MC2), indulging thoughts (MC1), and non-goal thoughts (MC0). For MC2, higher trait mindfulness significantly predicted a lower proportion of MC2 thoughts (β = −0.21, *SE* = 0.08, *z* = −2.58, *p* = .010). Neither depression nor anxiety significantly predicted MC2 proportions. No significant associations emerged for MC1 or MC0 with any well-being predictor (all *p*s > .39).

A follow-up operationalization restricting analyses to goal-related thoughts only (e.g. MC3 / MC1+MC2+MC3) also revealed consistent significant associations with all outcomes. This operationalization indexes depth of goal elaboration independent of frequency of engagement in goal-cognition. All primary well-being-MC3 found in the default analysis including all thoughts held in this operationalization: depression (β = −0.25, *SE* = 0.12, *p* = .031), anxiety (β = −0.29, *SE* = 0.11, *p* = .009), and mindfulness (β = 0.62, *SE* = 0.08, *p* < .001). Full analyses are reported in Supplemental Analysis S5.

### Discussion

Study 1 findings suggest that spontaneous mental contrasting during unprompted thought is a measurable phenomenon that meaningfully associates with indices of psychological well-being in young adults. Greater symptoms of depression and anxiety predicted less frequent MC3, while higher trait mindfulness predicted more frequent MC3 and less MC2. These foundational associations motivated Study 2, which aimed to replicate these findings in an independent sample while extending the analysis to include older adults and real-world goal attainment.

## Study 2

### Samples and Procedures

This pre-registered study^60^ was comprised of 95 adults including 49 younger adults (age range: 18–35, *M* = 22.7) and 46 older adults (age range: 60–84, *M* = 69.7). Participants were recruited via the University of Arizona Psychology website, social media advertisements, or referral from other studies and received cash compensation or e-gift card for participating. Written informed consent was obtained from all participants, and all procedures were approved by the University of Arizona’s Institutional Review Board. This sample identified as predominantly Non-Hispanic White (*n* = 70), with Latine/Hispanic (*n* = 13), and identifying as other racial or ethnic groups or preferring not to answer (*n* = 12). This study consisted of two remote sessions via Zoom during the COVID-19 pandemic. Each session lasted 60–90 minutes in length and were spaced 10 days apart.

### Scales and Measures

Participants completed self-report measures in Session 1, including the MAAS for mindfulness and the PHQ-9 for depressive symptoms. We did not use the GAD-7 as our anxiety measure in this study. Instead, we calculated a mean score using the 4-item Anxiety sub-scale score from the DSM-5 Self-Rated Level 1 Cross-Cutting Symptom Measure—Adult^69^ completed online. Participants reported mild-to-moderate depressive symptoms (*M* = 12.91, *SD* = 4.21 on the PHQ-9), mild anxiety (*M* = 5.10, *SD* = 2.42 on the DSM-5 Anxiety subscale), and moderate-to-high trait mindfulness (*M* = 4.28, *SD* = 0.74 on the MAAS).

#### Goal Elicitation, Characteristics, and Attainment

Participants completed a survey in Session 1 in which they provided four goals that they “would like to complete within the next 10 days,”, and provided a brief written description for each goal. They were also asked to report the degree to which their goal involved avoiding or approaching an outcome, the vividness of their mental imagery when imagining goal completion, similarity of each goal to past goals, and importance to self and others. At the end of Session 2, participants were presented with their brief description of each goal and reported their progress on each goal using a Likert scale from 1 (*no progress*) to 5 (*accomplished my goal*). They were additionally asked to rate the degree to which they had a plan or strategy in place to move towards each goal. See Supplementary Information for full descriptions of pre/post-task questions.

#### Think Aloud Protocol and Mental Contrasting Scoring

Participants completed a resting state TAP at the beginning of Session 2, approximately 10 days after Session 1, following the same procedures as Study 1 with the exception that the protocol was conducted via Zoom Health. Inter-rater reliability for MC score coding was excellent (κ*w* = 0.886, *z* = 36.80, *p* < .001). As the TAP was positioned approximately 10 days following goal elicitation, participants were likely not primed to think of their goals in the TAP paradigm.

### Results

Unless otherwise noted, analyses were pre-registered on OSF; exploratory analyses that were not part of the original analysis plan are labeled as such throughout. Variables were log-transformed and all regression analyses employed robust linear regression for the same reasons as in Study 1. On average, participants produced 16.8 thoughts (*SD* = 6.5, range: 5–38) during the TAP, fewer than Study 1 (*M* = 28.9, *SD* = 14.2). Younger and older adults produced comparable numbers of thoughts (YA: *M* = 17.9, *SD* = 7.2; OA: *M* = 15.6, *SD* = 5.5; *t*(93) = 1.69, *p* = .095). Approximately 34.6% of thoughts were goal-related (*M* = .37, *SD* = .19), notably higher than Study 1 (24.1%). Table 2 presents the full breakdown by age group.

#### Individual Difference Relationships between Mental Contrasting and Psychological Well-Being Largely Replicate in Study 2

**Fig. 3.**
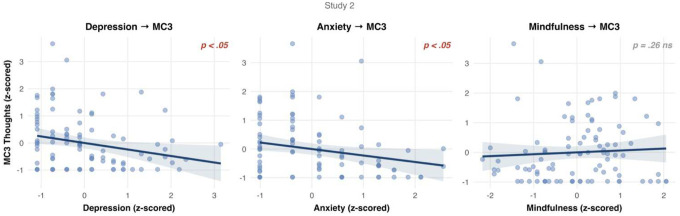
Relationships Between Well-Being and Spontaneous Goal Cognition (Study 2). Points represent individual participants. Lines reflect robust linear regression fits with 95% CI shading. Filled annotations indicate significant associations; gray annotations indicate marginal or non-significant.

We first assessed whether the associations between spontaneous MC and well-being observed in Study 1 replicated in the broader adult sample. Replicating Study 1, higher depressive symptomatology significantly predicted a lower proportion of MC3 thoughts (β = −0.22, *SE* = 0.09, *z* = −2.36, *p* = .018), and higher anxiety symptomatology similarly predicted lower MC3 (β = −0.21, *SE* = 0.09, *z* = −2.33, *p* = .020; Fig. 3). In contrast to Study 1 and our preregistered Hypotheses, trait mindfulness did not significantly predict MC3 or MC2, despite yielding parameter estimates in the same direction as Study 1 (MC3: β = 0.11, *p* = .257; MC2: β = −0.12, *p* = .134). Relationships with wellbeing attenuated (*ps* < .14) when restricting analyses to goal-related thoughts only (MC3 / MC1+MC2+MC3;Supplemental Analysis S5).

New relationships not observed in Study 1 emerged. Exploratory analyses revealed that higher depression also predicted greater MC2 dwelling thoughts (β = 0.20, *p* = .016) and marginally fewer MC1 indulging thoughts (β = −0.19, *p* = .059); anxiety and mindfulness did not significantly predict MC2 or MC1 (all *p*s > .13; full results in Supplemental Analysis S4b and Table S4).

Most relationships between MC level and wellbeing did not show an interaction with age group (all *p*s > .70), except for mindfulness, where among older adults, mindfulness predicted fewer dwelling thoughts MC2 (β = −0.35, *p* = .042) and more MC0 (β = 0.78, *p* < .001; see Supplementary Information). When age group was included as a covariate without an age interaction in mental health models, the depression-MC3 association attenuated to marginal-significance (β = −0.18, *SE* = 0.10, *z* = −1.71, *p* = .086), the anxiety-MC3 association similarly attenuated (β = −0.17, *SE* = 0.10, *z* = −1.76, *p* = .078), and the association with mindfulness remained non-significant (β = 0.04, *SE* = 0.10, *z* = 0.44, *p* = .657). This likely reflects shared variance between age and wellbeing; because older adults exhibit both higher wellbeing and MC3 (see below), age absorbed variance that is otherwise explained by wellbeing, reducing power to find effects when controlling for age (See Supplemental Analysis S5b for power analyses).

Given this pattern of findings, we pooled effect sizes across Study 1 and 2 following established procedures for combining correlations within a single analysis^70^ to examine whether associations between mental health traits and MC levels were statistically consistent across studies. For these analyses, Study 2 used effect sizes in models controlling for age group. Meta-analytic pooling confirmed consistent associations between depression and MC3 (*r* = −.27, 95% CI [−.41, −.13]), anxiety and MC3 (*r* = −.24, 95% CI [−.38, −.09]), and mindfulness and MC3 (*r* = .20, 95% CI [.05, .35]). Between-study heterogeneity tests revealed no significant differences for depression (*z* = −0.49, *p* = .624) or anxiety (*z* = −0.27, *p* = .790), supporting the conclusion that the well-being-MC3 associations are consistent and replicable across studies. Full meta-analytic results are reported in Supplemental Analysis S6.

#### Spontaneous Goal-Oriented Cognition and Wellbeing Differs between Older and Young Adults

**Fig. 4.**
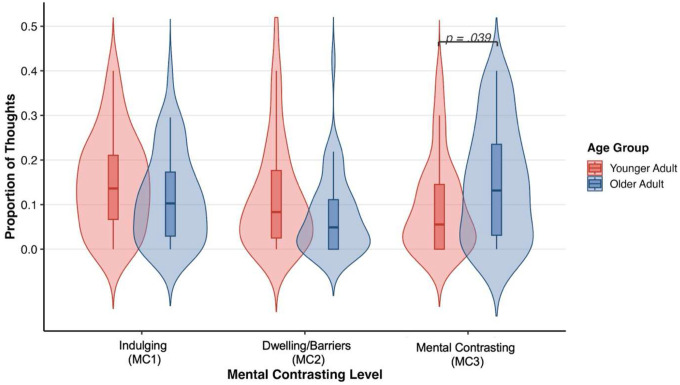
Age Differences in Mental Contrasting. Violin plots showing the distribution of MC thought proportions for younger adults and older adults at each MC level. Significance (p = .039) shown for the MC3 comparison.

Consistent with our preregistered Hypotheses, robust linear regression models confirmed that older adults produced a significantly higher proportion of MC3 thoughts compared to younger adults (M*OA* = 16.0%, M*YA* = 10.5%; β = 0.40, *SE* = 0.19, *z* = 2.07, *p* = .039; Table 2; Fig. 4). Older adults did not significantly differ from younger adults in their proportion of MC0 (t(93) = 1.69, p = .095), MC1 (*t*(93) = 1.59, *p* = .116), or MC2 (*t*(87.2) = 1.89, *p* = .062) thoughts. Older adults also demonstrated higher trait mindfulness (β = 0.67, *SE* = 0.21, *z* = 3.12, *p* = .002), lower depression (β = −0.53, *SE* = 0.19, *z* = −2.75, *p* = .006), and lower anxiety symptomatology (β = −0.65, *SE* = 0.23, *z* = −2.83, *p* = .005).

#### Groups Achieve Equivalent Goal Progress Despite Differences in Spontaneous Goal Cognition and Characteristics

Contrary to our Hypotheses, both age groups demonstrated comparable self-reported goal progress (M*OA* = 3.41, M*YA* = 3.46; *t*(91.3) = 0.33, *p* = .740) and number of goals accomplished (M*OA* = 1.22, M*YA* = 1.35; *t*(89.1) = 0.53, *p* = .600) across their four self-set goals over the 10-day period. Exploratory analyses of goal characteristics revealed significant age differences that contextualize these patterns. Older adults reported that their goals were more likely to incorporate a strategy to reach them (*p* < .001), were of greater personal and social importance (both *p*s < .05), and more avoidance-framed (*p* = .029), while younger adults reported more vivid goal imagery (*p* = .034). No age differences emerged for approach framing or goal progress (all *p*s > .36). Full results are presented in Supplemental Analysis S9 and Fig. S7.

#### Spontaneous Engagement in Mental Contrasting Predicts Real-World Goal Progress and Achievement

**Fig. 5.**
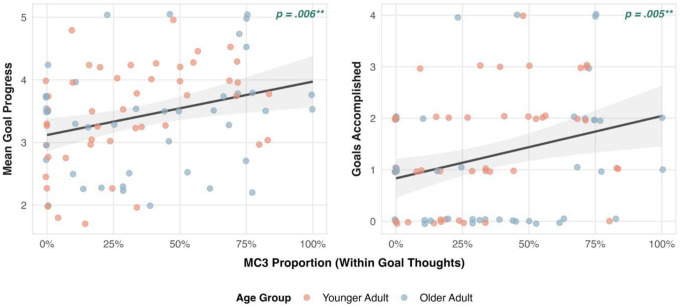
Spontaneous Mental Contrasting and Goal Attainment. MC3 as proportion of goal-related thoughts only across sample (n = 89). Points colored by age group. Lines represent OLS regression with 95% confidence intervals. Progress = mean Likert rating (1–5) across 4 goals. Accomplished = count of goals completed. ** p < .01.

Although overall frequency of MC3 did not significantly predict goal progress or goal achievement (β = 0.08, *p* = .400 and β = 0.07, *p* = .600, respectively), with or without controlling for age (both *p*s > .36), our follow-up operationalization restricting analyses to goal-related thoughts only (MC3 / MC1+MC2+MC3) revealed significant associations with both outcomes, consistent with the directions predicted in our preregistered Hypotheses. Within this operationalization, higher MC3 was significantly associated with greater mean goal progress (β = 0.25, *SE* = 0.09, *z* = 2.78, *p* = .006) and a higher count of goals accomplished (β = 0.34, *SE* = 0.12, *z* = 2.79, *p* = .005; Fig. 5), with both associations strengthening when controlling for age group (progress: β = 0.26, *SE* = 0.09, *z* = 2.85, *p* = .004; accomplishment: β = 0.37, *SE* = 0.12, *z* = 2.98, *p* = .003). Said differently, when individuals irrespective of age group thought about their goals, their propensity to think about their goals using MC3 strategies predicted both goal progress and goal achievement. See Supplemental Analysis S5 for the full set of follow-up goal progress models, including results using both operationalizations.

Consistent with preregistered Hypotheses, higher depressive symptoms significantly predicted lower goal progress (averaged across the four goals) both when age was (β = −0.22, *SE* = 0.10, *z* = −2.30, *p* = .021) and wasn’t (β = −0.21, SE = 0.10, z = −2.10, p = .036) included in the model. Anxiety and mindfulness did not significantly predict goal progress (both *p*s > .33). However, exploratory analyses showed that both depression and anxiety predicted fewer goals accomplished over the 10-day period (depression: β = −0.31, *p* = .008), while mindfulness predicted more (β = 0.29, *p* = .018).

## General Discussion

Findings across two studies (*N* = 173) position spontaneous, unprompted thought as a window into naturalistic goal pursuit and psychological well-being across the adult lifespan. Using a novel hierarchical scoring procedure (MC0-MC3), Study 1 established foundational associations between spontaneous MC and well-being in young adults.

Critically, these relationships emerged in the absence of any instruction or goal-relevant prompting, addressing a gap in the literature by providing estimates of how the hierarchy of FRT is naturally distributed across the unprompted stream of consciousness. Study 2 replicated many of these findings in a lifespan sample while additionally examining goal attainment and age-related differences in goal cognition. Together, these studies provide systematic evidence that individuals naturally engage in self-regulatory goal cognition during unprompted thought, that mentally contrasted goal thoughts (MC3) associate with better psychological health and greater goal success, and that these patterns differ meaningfully across the adult lifespan.

### Spontaneous Goal Elaboration Patterns Predict Psychological Well-Being

In Study 1, young adults with lower depression and anxiety and higher trait mindfulness spontaneously engaged in more complete mental contrasting. Several of these associations replicated in Study 2, and meta-analytic pooling confirmed consistent well-being-MC3 associations of small-to-medium magnitude across both studies.

The capacity to spontaneously self-generate adaptive regulatory strategies without external prompting may represent a robust indicator of self-regulatory skill and psychological resilience. Our findings are consistent with prior estimates that approximately 30–50% of resting-state thought involves goal-related content^50,52,71,72,73^ and extend them by characterizing how depth of goal-related thoughts are naturally distributed across levels of elaboration (MC1 – 3) in the stream of consciousness, and showing that individual variation in that depth relates meaningfully to psychological health.

Within our samples, goal-related thoughts were predominantly MC1 (*indulging: M =* 15.6%), followed by MC2 (*dwelling*: *M* = 4.1%) and MC3 (MC3: *M* = 4.4%). Critically, this extends prior work by Sevincer and Oettingen (Study 1 and 2)^53^, who provided the closest estimate of spontaneous MC prevalence (9–10%) in relation to writing about a desired wish. To our knowledge, the present studies are the first to address the prevalence of spontaneous MC in the absence of goal-related instruction or prompting.

The relationship between depression and incomplete goal elaboration (MC2) deserves attention. Across both studies, higher depression predicted greater dwelling-type thinking, elaborating on obstacles without identifying solutions, consistent with clinical characterizations of depression as rumination without constructive problem-solving^8,74,75^ and with evidence that depressed individuals anticipate less pleasure from goal attainment and greater distress from failure^16^. Depressed individuals may become stuck at obstacle awareness without progressing to solution-focused thinking, perpetuating passivity and possible disengagement over time.

Anxiety findings similarly support theoretical predictions. Higher anxiety predicted lower spontaneous MC3, consistent with anxiety’s avoidance-oriented motivational profile^12,13^. Anxious individuals’ tendency to generate negative consequences in response to potential goal non-attainment^17,76^ e.g., ‘what if they fire me for missing this deadline?’, may interfere with the obstacle-and-solution integration MC3 requires, promoting repetitive negative thinking and behavioral avoidance.

Mindfulness findings in Study 1 provided initial support for mindfulness as a protective factor in spontaneous goal cognition, with higher trait mindfulness predicting more complete MC and less dwelling. This association did not fully replicate in Study 2 when controlling for age. Supplementary interaction analyses revealed distinct pathways by age group: among younger adults, mindfulness was associated with more complete goal elaboration; among older adults, it was associated with fewer dwelling thoughts and a broader shift toward present-moment, non-goal-oriented awareness, consistent with theoretical accounts of mindfulness facilitating disengagement from goal-related rumination^19,20^ (see Supplementary Analyses S3). Importantly, meta-analytic results confirmed a consistent positive mindfulness-MC3 association across both studies, indicating that the Study 2 attenuation may reflect the finding that older adults reported higher mindfulness and higher MC3, such that controlling for age reduced the unique variance available for mindfulness to explain.

### Spontaneous Goal Cognition Reveals Greater Solution-Focused Elaboration in Older Adults

As predicted, older adults produced a significantly higher proportion of MC3 thoughts, revealing more solution-focused elaboration of one’s goals that was not present in younger adults. Exploratory analyses of goal characteristics further contextualize this pattern, as older adults were more likely to report having a strategy to reach their goals, rated their goals as higher in personal and social importance, and framed them more in avoidance terms, consistent with established lifespan theories such as SST^31,33^ and the SOC model^32^. These characteristics also align with broader developmental shifts from exploratory search towards exploitation of accumulated knowledge^35^, and with evidence that older adults prioritize maintenance and loss prevention over growth and gains, a pattern proposed to reflect adaptation rather than decline mechanisms in later life^2,3,36^. Older adults also demonstrated better psychological well-being alongside higher MC3 engagement. This raises an intriguing developmental question: does increased MC3 contribute to improved well-being with age, or does improved well-being enable more complete goal elaboration? If a causal relationship indeed exists, longitudinal research is needed to disentangle these directional relationships.

It is worth noting that this age-related MC3 pattern runs counter to predictions from several adjacent literatures. Age-related declines in executive function, reduced episodic specificity in future thinking^77^, and laboratory-based memory deficits^78^ would predict that older adults might struggle with the kind of integrated, multi-step elaboration MC3 requires. However, contrary to our predictions, both age groups demonstrated comparable goal progress and accomplishment over 10 days, and older adults showed no significant reduction in the total number of goal-related thoughts. This apparent paradox might reflect a broader pattern in cognitive aging research, whereby older adults’ cognitive profiles appear more adaptive in naturalistic, unprompted contexts than laboratory paradigms often suggest, a dissociation documented in the autobiographical ^memory79,80^, spontaneous thinking^81,82^, and naturalistic prospective memory literatures^83,84^. Consistent with this view, Turnbull and colleagues^85^ found that older adults demonstrate intact ability to adapt to different contexts and task focus (i.e., context regulation)^86^ under increasing demands in daily life. This pattern has been attributed to older adults developing less cognitively demanding but effective regulatory strategies over time^87^. The current findings suggest that what appears as cognitive decline under controlled conditions may reflect, at least in part, an age-related resource reallocation that becomes visible when thought unfolds without prompting or instruction.

### Spontaneous Mental Contrasting Predicts Real-World Goal Attainment

When individuals spontaneously engaged in goal-oriented thinking, higher mental contrasting was significantly associated with accomplishing and progressing towards more self-set goals over 10 days, which remained significant when controlling for age, establishing the ecological and functional relevance of naturally occurring MC. These findings extend prior theoretical accounts proposing that spontaneous goal-related thought during off-task periods may be adaptive^50,88^, showing that not all ways of thinking about one’s goals relate equally to goal attainment and underscoring the importance of examining not just whether goals are attained but how people cognitively navigate their pursuit.

### A Novel Method for Quantifying Spontaneous Goal-Related Thought

This research makes an important methodological contribution by adapting Fantasy Realization Theory’s constructs (FRT)^38^ to spontaneous thought via the Think-Aloud Protocol. The MC coding scheme (MC0-MC3) quantifies the depth of naturally occurring goal cognition without instruction or prompting, offering an ecologically valid window into everyday cognitive processes. The TAP occupies a middle ground between laboratory precision and the contextual richness of experience sampling, preserving the natural thought stream while enabling systematic analysis. The finding that approximately 27–38% of resting thought involves goal-related content provides an important benchmark consistent with prior frameworks^49,89^. Between-study variability in this proportion could reflect procedural context (Study 2) via Zoom Health, sample differences, and genuine individual and developmental variation.

### Limitations and Future Directions

Some limitations should be noted. First, our MC2 metric does not distinguish adaptive from maladaptive obstacle-related thinking, as described by FRT: a person who thoughtfully sets aside an unattainable goal receives the same code as one who ruminates unproductively; future work might incorporate complementary metrics such as thought length and repetitiveness to better distinguish these patterns. Second, both samples were cross-sectional, precluding causal conclusions about the direction of the MC3-well-being relationship, and reliance on a single 10-minute TAP session leaves open whether spontaneous MC3 reflects a stable trait or fluctuates with mood and context; repeated assessments and experience sampling would help resolve both questions. Third, goal attainment was assessed via short-term self-report rather than longer-term, objective outcomes, and samples were primarily well-functioning community adults, limiting generalizability to clinical populations where goal elaboration deficits may differ and where targeted interventions could prove valuable. Finally, the current paradigm cannot determine whether a given thought reflects deliberate or automatic cognition^90^; coding for repeated goal mentions or implementation-relevant content could more directly link elaboration depth to goal-directed behavior over time.

### Conclusions

Goals shape the landscape of human experience, providing direction, meaning, and structure to daily life. This research demonstrates that individuals naturally vary in the depth with which they elaborate on goals during unprompted thought, that these variations associate with mental health and goal success, and that these patterns differ across the adult lifespan in ways consistent with adaptive developmental processes. By bridging laboratory research with the naturalistic phenomenology of spontaneous thought, this work advances understanding of the cognitive processes supporting effective goal pursuit and psychological flourishing. The spontaneous generation of solution-focused goal elaboration may represent a measurable cognitive skill underlying successful adaptation to life’s challenges.

## Supplementary Files

This is a list of supplementary files associated with this preprint. Click to download.

- PuigRiveraetalgoalssuppmatv6260620finalsubmitted.pdf

## Figures and Tables

**Table 1.** Study 1 Descriptive Statistics: Thought Distribution and MC Level Counts

Study 1 (n = 77)			
Parameter	M (SD) [%]	Median	Range
**Total Thoughts**	28.9 (14.2)	28	5 – 63
**Goal-Related Thoughts (MC1–MC3)**	8.1 (5.2) [24.1%]	8	0 – 22
** MC Level 0 (Non-Goal)**	21.8 (12.9) [75.9%]	20	1 – 59
** MC Level 1 (Indulging)**	5.1 (4) [15.6%]	4	0 – 18
** MC Level 2 (Dwelling)**	2.1 (2.3) [4.1%]	1	0 – 11
** MC Level 3 (Contrasting)**	1 (1.6) [4.4%]	0	0 – 7

Note. Values reflect mean raw count (SD) with mean group percentage in brackets. Goal-Related Thoughts = MCI + MC2 + MC3.

**Table 2.** Study 2 Descriptive Statistics: Thought Distribution and MC Level Counts

	Full Sample (N = 95)			Younger Adults (n = 49)			Older Adults (n = 46)		
Parameter	M (SD) [%]	Median	Range	M (SD) [%]	Median	Range	M (SD) [%]	Median	Range
**Total Thoughts**	16.8 (6.5)	16	5 – 38	17.9 (7.2)	17	5 – 38	15.6 (5.5)	16	6 – 27
**Goal-Related Thoughts (MC1-MC3)**	6.6 (4.6) [34.6%]	6	0 – 18	7.4 (4.7) [35.6%]	6.5	0 – 18	5.8 (4.3) [33.7%]	5.2	0 – 18
** MC Level 0 (Non-Goal)**	10.1 (3.7) [64%]	10	2 – 20	10.4 (4.4) [63%]	10.5	2 – 20	9.9 (3) [65.1%]	10	2 – 17
** MC Level 1 (Indulging)**	2.5 (2.7) [13.2%]	2	0 – 12	3 (3) [14.8%]	2.5	0 – 12	1.9 (2.1) [11.4%]	1.5	0 – 10
** MC Level 2 (Dwelling)**	1.7 (1.8) [9.7%]	1	0 – 8	2 (1.8) [11.6%]	1.5	0 – 6	1.3 (1.7) [7.6%]	1	0 – 8
** MC Level 3 (Contrasting)**	2.4 (2.5) [13.2%]	1.5	0 – 12	2.3 (2.6) [10.5%]	1	0 – 12	2.6 (2.4) [16%]	2.2	0 – 9

Note. Values reflect mean raw count (SD) with mean group percentage in brackets. Percentage not shown for Total Thoughts. Goal-Related Thoughts = MCI + MC2 + MC3. Mdn = median.

## References

[1] Diener E., & Larsen R. J. (1993). The experience of emotional well-being. In Lewis M. & Haviland J. M. (Eds.), Handbook of emotions (pp. 405–415). Guilford Press.

[2] Ebner N. C., Freund A. M., & Baltes P. B. (2006). Developmental Changes in Personal Goal Orientation From Young to Late Adulthood: From Striving for Gains to Maintenance and Prevention of Losses. Psychology and Aging, 21(4), 664–678. 10.1037/0882-7974.21.4.664 17201488

[3] Freund A. M. (2024). Goals in old age: What we want when we are old and why it matters. Current Opinion in Psychology, 57, Article 101803. 10.1016/j.copsyc.2024.101803 38432188

[4] MacLeod A. (2013). Goals and Plans: Their Relationship to Well-Being. In Moraitou D. & Efklides A. (Eds.), A Positive Psychology Perspective on Quality of Life (pp. 33–50). Springer Netherlands. 10.1007/978-94-007-4963-4_3

[5] Wrosch C., & Scheier M. F. (2020). Adaptive self-regulation, subjective well-being, and physical health: The importance of goal adjustment capacities. In *Advances in motivation science*(Vol. 7, pp. 199–238). Elsevier. 10.1016/bs.adms.2019.07.001

[6] Diener E., Kesebir P., & Tov W. (2009). Happiness. In Leary M. & Hoyle R. (Eds.), Handbook of individual differences in social behavior (pp. 147–160). Guilford Press.

[7] Freund A. M., & Baltes P. B. (2002). The Adaptiveness of Selection, Optimization, and Compensation as Strategies of Life Management: Evidence From a Preference Study on Proverbs. The Journals of Gerontology. Series B, Psychological Sciences and Social Sciences, 57(5), P426–P434. 10.1093/geronb/57.5.P426 12198101

[8] Gamble B., Tippett L. J., Moreau D., & Addis D. R. (2021). The Futures We Want: How Goal-Directed Imagination Relates to Mental Health. Clinical Psychological Science, 9(4), Article 2167702620986096. 10.1177/2167702620986096

[9] Sheldon K. M., Kasser T., Smith K., & Share T. (2002). Personal Goals and Psychological Growth: Testing an Intervention to Enhance Goal Attainment and Personality Integration. Journal of Personality, 70(1), 5–31. 10.1111/1467-6494.00176 11908535

[10] Klinger E., & Cox W. M. (2011). Motivation and the Goal Theory of Current Concerns. In Klinger E. & Cox W. M. (Eds.), Handbook of Motivational Counseling: Goal-Based Approaches to Assessment and Intervention with Addiction and Other Problems (pp. 1–47). John Wiley & Sons, Ltd. 10.1002/9780470979952.ch1

[11] Dickson J. M., & MacLeod A. K. (2004a). Anxiety, depression and approach and avoidance goals. Cognition and Emotion, 18(3), 423–430. 10.1080/02699930341000013

[12] Dickson J. M., & MacLeod A. K. (2004b). Approach and Avoidance Goals and Plans: Their Relationship to Anxiety and Depression. Cognitive Therapy and Research, 28(3), 415–432. 10.1023/B:COTR.0000031809.20488.ee

[13] Winch A., Moberly N. J., & Dickson J. M. (2015). Unique associations between anxiety, depression and motives for approach and avoidance goal pursuit. Cognition & Emotion, 29(7), 1295–1305. 10.1080/02699931.2014.976544. 25379697

[14] Street H. (2002). Exploring Relationships Between Goal Setting, Goal Pursuit and Depression: A Review. Australian Psychologist, 37(2), 95–103. 10.1080/00050060210001706736

[15] Dickson J. M., Moberly N. J., & Kinderman P. (2011). Depressed people are not less motivated by personal goals but are more pessimistic about attaining them. Journal of Abnormal Psychology, 120(4), 975–980. https://doi-org.ezproxy3.library.arizona.edu/10.1037/a0023665 21553938 10.1037/a0023665

[16] MacLeod A. K., & Salaminiou E. (2001). Reduced positive future-thinking in depression: Cognitive and affective factors. Cognition and Emotion, 15(1), 99–107. 10.1080/02699930125776

[17] MacLeod A. K., Tata P., Kentish J., Carroll F., & Hunter E. (1997). Anxiety, Depression, and Explanation-based Pessimism for Future Positive and Negative Events. Clinical Psychology and Psychotherapy, 4(1), 15–24. https://doi-org.ezproxy4.library.arizona.edu/10.1002/(SICI)1099-0879(199703)4:1<15::AID-CPP112>3.0.CO;2-#

[18] Brown K. W., & Ryan R. M. (2003). The Benefits of Being Present: Mindfulness and Its Role in Psychological Well-Being. Journal of Personality and Social Psychology, 84(4), 822–848. 10.1037/0022-3514.84.4.822 12703651

[19] Kabat-Zinn J. (1994). Wherever you go, there you are: Mindfulness meditation in everyday life. Hyperion. New York (1994). xxi + 277. Behaviour Research and Therapy, 33(8), 996–996. 10.1016/0005-7967(95)90133-7

[20] Levesque C., & Brown K. W. (2007). Mindfulness as a moderator of the effect of implicit motivational self-concept on day-to-day behavioral motivation. Motivation and Emotion, 31(4), 284–299. 10.1007/s11031-007-9075-8

[21] Sheldon K. M., & Elliot A. J. (1999). Goal Striving, Need Satisfaction, and Longitudinal Well-Being: The Self-Concordance Model. Journal of Personality and Social Psychology, 76(3), 482–497. 10.1037/0022-3514.76.3.482 10101878

[22] Hoemann K., Lee Y., Kuppens P., Gendron M., & Boyd R. L. (2023). Emotional granularity is associated with daily experiential diversity. Affective Science, 4(2), 291–306. 10.1007/s42761-023-00185-2. 37304562 PMC10247944

[23] Van der Gucht K., Dejonckheere E., Erbas Y., Takano K., Vandemoortele M., Maex E., Raes F., & Kuppens P. (2019). An experience sampling study examining the potential impact of a mindfulness-based intervention on emotion differentiation. Emotion, 19(1), 123–131. https://doi-org.ezproxy2.library.arizona.edu/10.1037/emo0000406 29578747 10.1037/emo0000406

[24] Chambers R., Gullone E., & Allen N. B. (2009). Mindful emotion regulation: An integrative review. Clinical Psychology Review, 29(6), 560–572. 10.1016/j.cpr.2009.06.005 19632752

[25] King A. P., & Fresco D. M. (2019). A neurobehavioral account for decentering as the salve for the distressed mind. Current Opinion in Psychology, 28, 285–293. 10.1016/j.copsyc.2019.02.009 31059966 PMC6706318

[26] SgherzaT. R., BecerraR., BylsmaL. M., FrescoD. M., & Naragon-GaineyK. (2026). The protective effect of decentering on the links between internalising symptoms, functional impairment and wellbeing. Behavior Therapy. 10.1016/j.beth.2026.04.00242660582

[27] Dominguez E., Casagrande M., & Raffone A. (2022). Autobiographical memory and mindfulness: A critical review with a systematic search. Mindfulness, 13(7), 1614–1651. 10.1007/s12671-022-01902-x

[28] Brandtstädter J & Rothermund K. (2002). The life-course dynamics of goal-pursuit and goal adjustment: a two-day process framework. Developmental Review. 22, 117 – 150. 10.1006/drev.2001.0539

[29] Buchinger L., Richter D., & Heckhausen J. (2022). The Development of Life Goals Across the Adult Life Span. The Journals of Gerontology. Series B, Psychological Sciences and Social Sciences, 77(5), 905–915. 10.1093/geronb/gbab154 34417801

[30] Carstensen L. L. (2021). Socioemotional Selectivity Theory: The Role of Perceived Endings in Human Motivation. The Gerontologist, 61(8), 1188–1196. 10.1093/geront/gnab116 34718558 PMC8599276

[31] Carstensen L. L., Isaacowitz D. M., & Charles S. T. (1999). Taking Time Seriously: A Theory of Socioemotional Selectivity. The American Psychologist, 54(3), 165–181. 10.1037/0003-066X.54.3.165 10199217

[32] Carstensen L. L., & Reynolds M. E. (2023). Age differences in preferences through the lens of socioemotional selectivity theory. The Journal of the Economics of Ageing, 24. 10.1016/j.jeoa.2022.100440

[33] Baltes P. B., & Baltes M. M. (1990). Psychological perspectives on successful aging: The model of selective optimization with compensation. In Baltes P. B. & Baltes M. M. (Eds.), Successful aging: Perspectives from the behavioral sciences (Vol. 1, pp. 1–34). Cambridge University Press.

[34] Baltes P. B., & Carstensen L. L. (1996). The process of successful ageing. Ageing and Society, 16(4), 397–422. 10.1017/S0144686X00003603

[35] Spreng R. N., & Turner G. R. (2021). From exploration to exploitation: a shifting mental mode in late life development. Trends in Cognitive Sciences, 25(12), 1058–1071. 10.1016/j.tics.2021.09.001 34593321 PMC8844884

[36] Heckhausen J., Brandstätter V., Fishbach A., Freund A. M., Lachman M. E., & Robert P. (2021). Goal Changes and Healthy Aging. The Journals of Gerontology. Series B, Psychological Sciences and Social Sciences, 76(Supplement_2), S105–S114. 10.1093/geronb/gbab038 34515773

[37] Oettingen G., Pak H., & Schnetter K. (2001). Self-Regulation of Goal Setting: Turning Free Fantasies About the Future Into Binding Goals. Journal of Personality and Social Psychology, 80(5), 736–753. 10.1037/0022-3514.80.5.736 11374746

[38] Oettingen G. (2000). Expectancy effects on behavior depend on self-regulatory thought. Social Cognition, 18(2), 101–129. 10.1521/soco.2000.18.2.101

[39] Oettingen G. (2012). Future thought and behaviour change. European Review of Social Psychology, 23, 1–63. doi:10.1080/10463283.2011.643698

[40] Oettingen G., Marquardt M. K., & Gollwitzer P. M. (2012). Mental contrasting turns positive feedback on creative potential into successful performance. Journal of Experimental Social Psychology, 48(5), 990–996. 10.1016/j.jesp.2012.03.008

[41] Marquardt M. K., Oettingen G., Gollwitzer P. M., Sheeran P., & Liepert J. (2017). Mental Contrasting With Implementation Intentions (MCII) Improves Physical Activity and Weight Loss Among Stroke Survivors Over One Year. Rehabilitation Psychology, 62(4), 580–590. 10.1037/rep0000104 29265873

[42] Oettingen G., Kappes H. B., Guttenberg K. B., & Gollwitzer P. M. (2015). Self-regulation of time management: Mental contrasting with implementation intentions. European Journal of Social Psychology, 45(2), 218–229. 10.1002/ejsp.2090

[43] Oettingen G. (2026). From conscious wishes to non-conscious action. In Vail K., Van Tongeren D., Schlegel R., Greenberg J., King L., & Ryan R. M. (Eds.), Handbook of the science of existential psychology (pp. 727–742). Guilford Press.

[44] Kappes A., Singmann H., & Oettingen G. (2012). Mental contrasting instigates goal pursuit by linking obstacles of reality with instrumental behavior. Journal of Experimental Social Psychology, 48(4), 811–818. 10.1016/j.jesp.2012.02.002

[45] Kappes A., & Oettingen G. (2014). The emergence of goal pursuit: Mental contrasting connects future and reality. Journal of Experimental Social Psychology, 54, 25–39. doi:10.1016/j.jesp.2014.03.014

[46] Gilles A., Panneels G., D’Argembeau A., & Stawarczyk D. (2025). Validity of the think-aloud procedure in comparison to other methods for studying the phenomenological features and memory of spontaneous thought. Consciousness and Cognition, 134, Article 103910. 10.1016/j.concog.2025.103910 40753816

[47] McVay J. C., Kane M. J., & Kwapil T. R. (2009). Tracking the train of thought from the laboratory into everyday life: An experience-sampling study of mind wandering across controlled and ecological contexts. Psychonomic bulletin & review, 16(5), 857–863. 10.3758/PBR.16.5.857 19815789 PMC2760023

[48] Kane M. J., Gross G. M., Chun C. A., Smeekens B. A., Meier M. E., Silvia P. J., & Kwapil T. R. (2017). For whom the mind wanders, and when, varies across laboratory and daily-life settings. Psychological science, 28(9), 1271–1289. 10.1177/0956797617706086 28719760 PMC5591044

[49] Klinger E. (2013). Goal Commitments and the content of thoughts and dreams: basic principles. Frontiers in Psychology, 4, Article Article 415. 10.3389/fpsyg.2013.00415 PMC3708449 23874312

[50] Mildner J. N., & Tamir D. I. (2024). Why do we think? The dynamics of spontaneous thought reveal its functions. PNAS Nexus, 3(6), Article pgae230. 10.1093/pnasnexus/pgae230 PMC11210302 38939015

[51] Stawarczyk D., Cassol H., & D’Argembeau A. (2013). Phenomenology of future-oriented mind-wandering episodes. Frontiers in Psychology, 4, Article Article 425. 10.3389/fpsyg.2013.00425 PMC3712143 23882236

[52] Andrews-Hanna J. R., Reidler J. S., Huang C., & Buckner R. L. (2010). Evidence for the default network’s role in spontaneous cognition. Journal of Neurophysiology, 104(1), 322–335. 10.1152/jn.00830.2009 20463201 PMC2904225

[53] Sevincer A. T., & Oettingen G. (2013). Spontaneous Mental Contrasting and Selective Goal Pursuit. Personality & Social Psychology Bulletin, 39(9), 1240–1254. 10.1177/0146167213492428 23831856

[54] Sevincer A. T., Mehl P. J., & Oettingen G. (2017). Well Self-Regulated People Use Mental Contrasting. Social Psychology (Göttingen, Germany), 48(6), 348–364. 10.1027/1864-9335/a000322

[55] James W. (2008). The stream of consciousness. Modernism: Critical Concepts in Literary and Cultural Studies, 1, 1890–1934.

[56] Wolcott M. D., & Lobczowski N. G. (2021). Using cognitive interviews and think-aloud protocols to understand thought processes. Currents in Pharmacy Teaching and Learning, 13(2), 181–188. 10.1016/j.cptl.2020.09.005 33454077

[57] Yu Y., Raffaelli Q., Freveletti D., Brossart J. J., Andrews E., Wilcox R. R., Grilli M. D., & Andrews-Hanna J. R. (2025). Mapping content and dynamics in the stream of consciousness through latent brain state analysis. iScience, 28(11), Article 113755. 10.1016/j.isci.2025.113755 PMC12682288 41362768

[58] Raffaelli Q., Malusa R., de Stefano N. A., Andrews E., Grilli M. D., Mills C., … & Andrews-Hanna J. R. (2024). Creative minds at rest: creative individuals are more associative and engaged with their idle thoughts. Creativity research journal, 36(3), 396–412. 10.1080/10400419.2023.2227477 39132452 PMC11315452

[59] Raffaelli Q., Mills C., de Stefano N.-A., Mehl M. R., Chambers K., Fitzgerald S. A., Wilcox R., Christoff K., Andrews E. S., Grilli M. D., O’Connor M.-F., & Andrews-Hanna J. R. (2021). The think aloud paradigm reveals differences in the content, dynamics and conceptual scope of resting state thought in trait brooding. Scientific Reports, 11(1), Article 19362. 10.1038/s41598-021-98138-x PMC8484343 34593842

[60] Puig Rivera V. A., Andrews-Hanna J. R., & Grilli M. D. (2024, February 1). Exploring relationships between goal-oriented cognition, psychological well-being and progression towards one’s goals. 10.17605/OSF.IO/6J2W7

[61] Kroenke K., R. L., & Williams J. B. W. (2001). The PHQ-9: Validity of a brief depression severity measure. Journal of General Internal Medicine : JGIM, 16(9), 606–613. 10.1046/j.1525-1497.2001.016009606.x 11556941 PMC1495268

[62] Spitzer R. L., Kroenke K., Williams J. B. W., & Löwe B. (2006). A Brief Measure for Assessing Generalized Anxiety Disorder: The GAD-7. Archives of Internal Medicine (1960), 166(10), 1092–1097. 10.1001/archinte.166.10.1092 16717171

[63] Ericsson K. A., & Simon H. A. (1993). Protocol analysis : verbal reports as data (2nd ed.). MIT Press. 10.7551/mitpress/5657.001.0001

[64] Sripada C., & Taxali A. (2020). Structure in the stream of consciousness: Evidence from a verbalized thought protocol and automated text analytic methods. Consciousness and Cognition, 85, Article 103007. 10.1016/j.concog.2020.103007 32977240

[65] Landis J. R., & Koch G. G. (1977). The Measurement of Observer Agreement for Categorical Data. Biometrics, 33(1), 159–174. 10.2307/2529310 843571

[66] Maechler M., Rousseeuw P., Croux C., Todorov V., Ruckstuhl A., Salibián-Barrera M., … di Palma M. A. (2023). robustbase: Basic robust statistics. R package version 0.99–0.

[67] Zeileis A., & Hothorn T. (2002). lmtest: Testing linear regression models. R package version 0.9–40.

[68] R Core Team (2026). _R: A Language and Environment for Statistical Computing_. R Foundation for Statistical Computing, Vienna, Austria. https://www.R-project.org/

[69] Narrow W. E., Clarke D. E., Kuramoto S. J., Kraemer H. C., Kupfer D. J., Greiner L., & Regier D. A. (2013). DSM-5 Field Trials in the United States and Canada, Part III: Development and Reliability Testing of a Cross-Cutting Symptom Assessment for DSM-5. The American Journal of Psychiatry, 170(1), 71–82. 10.1176/appi.ajp.2012.12071000 23111499

[70] Goh J. X., Hall J. A., & Rosenthal R. (2016). Mini meta-analysis of your own studies: Some arguments on why and a primer on how. Social and Personality Psychology Compass, 10(10), 535–549. 10.1111/spc3.12267

[71] D’Argembeau A. (2016). The Role of Personal Goals in Future-Oriented Mental Time Travel. In Seeing the Future. Oxford University Press. 10.1093/acprof:oso/9780190241537.003.0010.

[72] D’Argembeau A., & Mathy A. (2011). Tracking the Construction of Episodic Future Thoughts. Journal of Experimental Psychology. General, 140(2), 258–271. 10.1037/a0022581 21401291

[73] Fox K. C., & Beaty R. E. (2019). Mind-wandering as creative thinking: neural, psychological, and theoretical considerations. Current Opinion in Behavioral Sciences, 27, 123–130. 10.1016/j.cobeha.2018.10.009

[74] Anderson R. J., McClure J. H., Boland J., Howe D., Riggs K. J., & Dewhurst S. A. (2023). The relationship between depressive symptoms and positive emotional anticipation of goal achievement. Journal of Experimental Psychopathology, 14(1). 10.1177/20438087231164963

[75] Nolen-Hoeksema S. (2000). The role of rumination in depressive disorders and mixed anxiety/depressive symptoms. Journal of Abnormal Psychology, 109(3), 504–511. 10.1037/0021-843X.109.3.504 11016119

[76] MacLeod A. K. (2025). Future-directed thinking and emotional disorder. Journal of Applied Research in Memory and Cognition, 14(1), 1–14. 10.1037/mac0000211

[77] Schacter D. L., Gaesser B., & Addis D. R. (2013). Remembering the past and imagining the future in the elderly. Gerontology, 59(2), 143–151. 10.1159/000342198 22987157 PMC3645892

[78] Simpson S., Eskandaripour M., & Levine B. (2023). Effects of healthy and neuropathological aging on autobiographical memory: A meta-analysis of studies using the autobiographical interview. The Journals of Gerontology: Series B, 78(10), 1617–1624. 10.1093/geronb/gbad077 PMC10561892 37224530

[79] Puig Rivera V. A., Andrews E., Cervantes L. J., Freveletti D., Huentelman M., Grilli M. D., & Andrews-Hanna J. R. (2026). Enhanced episodic specificity and socioemotional content in older adults’ everyday autobiographical thoughts. Proceedings of the National Academy of Sciences - PNAS, 123(2), Article e2513990123. 10.1073/pnas.2513990123 PMC12799099 41505522

[80] McVeigh K. S., Deffner A. M., Hernandez D. A., Sbarra D. A., Mehl M. R., Andrews-Hanna J. R., & Grilli M. D. (in press). In here and out there: Evidence that age-related differences in memory specificity are attenuated in natural social conversations. Journal of Experimental Psychology: General. 10.31234/osf.io/sja6t_v2 PMC13425904 42531004

[81] Jordão M, Pinho M.S., St. Jacques P.L. (2020) The effects of aging and an episodic specificity induction on spontaneous task-unrelated thought. PLoS ONE 15(8): e0237340. 10.1371/journal.pone.0237340 32776948 PMC7416953

[82] Warden E.A., Plimpton B. & Kvavilashvili L. Absence of age effects on spontaneous past and future thinking in daily life. Psychological Research 83, 727–746 (2019). 10.1007/s00426-018-1103-7 30269274

[83] Haines S. J., Randall S. E., Terrett G., Busija L., Tatangelo G., McLennan S. N., Rose N. S., Kliegel M., Henry J. D., & Rendell P. G. (2020). Differences in time-based task characteristics help to explain the age-prospective memory paradox. Cognition, 202. 10.1016/j.cognition.2020.104305/32497925

[84] Maillet D., & Schacter D. L. (2016). From mind wandering to involuntary retrieval: Age-related differences in spontaneous cognitive processes. Neuropsychologia, 80, 142–156. 10.1016/j.neuropsychologia.2015.11.017 26617263 PMC4698179

[85] Turnbull A., Poerio G. L., Ho N. S., Martinon L. M., Riby L. M., Lin F. V., … & Smallwood J. (2021). Age-related changes in ongoing thought relate to external context and individual cognition. Consciousness and cognition, 96. 10.1016/j.concog.2021.103226 34689074

[86] Smallwood J., & Schooler J. W. (2015). The Science of Mind Wandering: Empirically Navigating the Stream of Consciousness. Annu. Rev. Psychol, 66, 487–518. 10.1146/annurev-psych-010814-015331 25293689

[87] Mata R., Schooler L. J., & Rieskamp J. (2007). The aging decision maker: cognitive aging and the adaptive selection of decision strategies. Psychology and aging, 22(4), 796. DOI: 10.1037/0882-7974.22.4.796 18179298

[88] Smallwood J., & Andrews-Hanna J. (2013). Not all minds that wander are lost: the importance of a balanced perspective on the mind-wandering state. Frontiers in psychology, 4, 441. 10.3389/fpsyg.2013.00441 23966961 PMC3744871

[89] Christoff K., Gordon A., & Smith R. (2011). The role of spontaneous thought in human cognition. In Mandel D. R. & Vartanian O. (Eds.), Neuroscience of Decision Making (1st ed., pp. 259–284). Psychology Press. 10.4324/9780203835920-17

[90] Christoff K., Irving Z., Fox K. Mind-wandering as spontaneous thought: a dynamic framework. Nat Rev Neurosci 17, 718–731 (2016). 10.1038/nrn.2016.113 27654862

